# Post-Glycosylation Modification of Sialic Acid and Its Role in Virus Pathogenesis

**DOI:** 10.3390/vaccines7040171

**Published:** 2019-11-01

**Authors:** Simon S. Park

**Affiliations:** 1 Department of Surgery, Beth Israel Deaconess Medical Center, Harvard Medical School, 330 Brookline Avenue Boston, MA 02215, USA; spark9@bidmc.harvard.edu; 2 Wyss Institute for Biologically Inspired Engineering, Harvard University, Center for Life Sciences 11096, 3 Blackfan Circle, MA 02215, USA

**Keywords:** sialic acid, vaccine targets, post-glycosylation modification, influenza, carbohydrate-based therapeutics, *O*-acetylation

## Abstract

Sialic acids are a family of nine carbon keto-aldononulosonic acids presented at the terminal ends of glycans on cellular membranes. α-Linked sialoglycoconjugates often undergo post-glycosylation modifications, among which *O*-acetylation of *N*-acetyl neuraminic acid (Neu5Ac) is the most common in mammalian cells. Isoforms of sialic acid are critical determinants of virus pathogenesis. To date, the focus of viral receptor-mediated attachment has been on Neu5Ac. *O*-Acetylated Neu5Acs have been largely ignored as receptor determinants of virus pathogenesis, although it is ubiquitous across species. Significantly, the array of structures resulting from site-specific *O*-acetylation by sialic acid *O*-acetyltransferases (SOATs) provides a means to examine specificity of viral binding to host cells. Specifically, C_4_ *O*-acetylated Neu5Ac can influence virus pathogenicity. However, the biological implications of only *O*-acetylated Neu5Ac at C_7–9_ have been explored extensively. This review will highlight the biological significance, extraction methods, and synthetic modifications of C_4_ *O*-acetylated Neu5Ac that may provide value in therapeutic developments and targets to prevent virus related diseases.

## 1. Introduction

**Figure 1:**
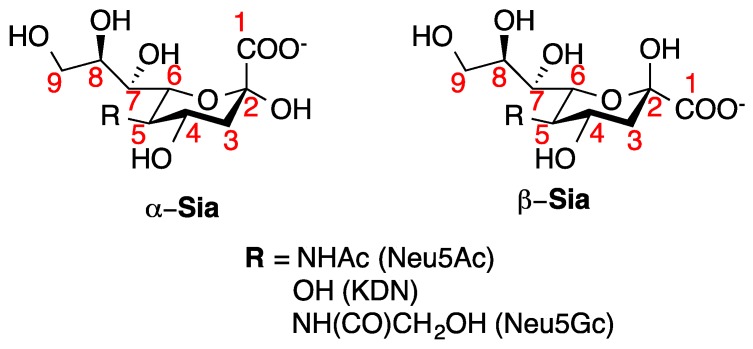
Examples of the structurally diverse sialic acid family.

The discovery of sialic acid [1] from bovine submaxillary mucin in 1936 gave the first glance to a unique family of sugars that play important roles in a wide array of biological functions. Currently, sialic acid (Sia) refers to a family of more than 50 forms typically varying at the C_5_ position (Figure 1). Chief among these in mammalian cells [2] is *N*-acetyl neuraminic acid (Neu5Ac), which is a keto-aldononulosonic acid positioned at the terminal ends of glycolipids and glycoproteins [3].

Sialyltransferases are responsible primarily for installing terminal Sia to glycoconjugates with defined glycosidic linkages using cytidine 5′-monophosphate (CMP) sialic acid donors. The nature of the glycosidic linkages involving sialic acid depends on the acceptors. Sialoglycoconjugates commonly are linked α-2,3 or α-2,6 to galactose or *N*-acetyl galactosamine, while polymeric forms of sialylation renders α-2,8 or α-2,9 glycosidic linkages [4,5,6,7,8,9,10]. Post-glycosylation, these sialoglycoconjugates are often modified and *O*-acetylation is the most common form in mammalian cells [11]. Additional forms of post-glycosylation modifications that increase Sia diversity include: *O*-methylation, sulfation, phosphorylation, and lactylation [12]. 

While *O*-acetylated forms of Sia are ubiquitous in mammalian cells, association of structure-to-function remains incomplete. Challenges in isolations and characterizations of *O*-acetylated Sias lead to insufficient amount of materials that limit biologically meaningful studies. Additionally, *O*-acetylation often leads to acetyl migrations between proximal hydroxyl positions and adds further complexity to the study of these compounds. Therefore, generation of synthetic standards in high purity could serve as useful tools to probe the functional significance of *O*-acetylated Sia [13,14]. 

*O*-Acetylation can occur in mono-, di-, tri-, or tetra- forms at varying positions of Neu5Ac. Using gas-chromatography mass spectrometry (GC-MS) and nuclear magnetic resonance (NMR) spectroscopy, a series of *O*-acetylated sialoglycoconjugates are extracted and identified across species (Table 1). The most common forms of *O*-acetylation across species occur at C_7_,_8_, and _9_ positions; however, *O*-acetylation at the C_4_ directly attached to the pyranosyl ring is also feasible. 

**Figure 2:**
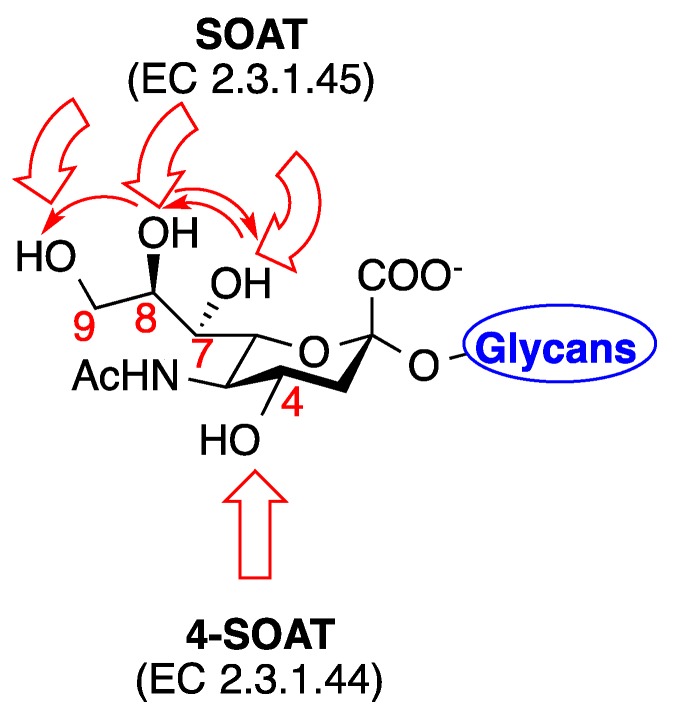
SOATs responsible for *O*-acetylation at specific sites of Neu5Ac. *O*-Acetylation by SOAT (EC 2.3.1.45) occurs at the C_7_–_9_ and acetyl migration is common. SOAT (EC 2.3.1.44) is responsible for *O*-acetylation at the C_4_ [26,27,28].

Sialic acid-*O*-acetyltransferases (SOATs) are responsible for site-specific *O*-acetylation of Neu5Ac [15,16,17,18]. SOATs *O*-acetylate at two distinct regions of Neu5Ac: SOAT (EC 2.3.1.45) [19,20] is primarily responsible for *O*-acetylation on the extended alkyl chain of Sia at C_7–9_ while 4-SOAT (EC 2.3.1.44) is responsible for *O*-acetylation at the C_4_ position on the pyranosyl ring (Figure 2) [21,22,23]. Although SOAT (EC 2.3.1.45) has been well documented for its structural and biological roles, purification and isolation of 4-SOAT (EC 2.3.1.44) has been unsuccessful [23] and studies related to C_4_-*O*-acetylated Neu5Ac remain poorly understood.

While SOATs are selective, acetyl migration within the extended alkyl chain is not uncommon, leading to diverse *O*-acetyl forms at C_7–9_. Schauer and co-workers showed that SOAT (EC 2.3.1.45) incorporates radioactively labeled acetyl CoA exclusively at the C_7_ hydroxyl position of Neu5Ac in bovine submandibular glands [24]; however, prolonged incubation time led to the formation of Neu5,9Ac_2_. Vliegenthart and co-workers also observed in another study, *O*-acetyl migration from C_7_ to C_9_ of Neu5Ac in bovine submandibular gland glycoproteins using ^1^H NMR [25]. 

*O*-Acetyl migration in bacterial systems, however, is more promiscuous and less predictable. Regarding *N. meningitidis*, acetyl migration of Type C polysaccharide occurs from C_8_ to C_7_ when monitored by ^1^H and ^13^C NMR [26,27], while acetyl groups of serogroup W-135 polysaccharides containing α-(2,6)-linkages to galactose or glucose spontaneously migrate from C_7_ to C_9_ [28]. As a result, it was believed that C_9_ *O*-acetylated Neu5Ac is a product of acetyl migration, although Varki and co-workers speculated that sialoglycoconjugates in the Golgi may be *O*-acetylated exclusively at C_7_ and C_9_ by different SOATs [29]. More recently, Gilbert and co-workers supported the idea, through 1D and 2D NMR studies, that SOATs identified from *C. jejuni* can exclusively *O*-acetylate at the C_9_ hydroxyl group [18]. These studies suggest that acetyl migrations between proximal hydroxyl groups at C_7_ (or C_8_) and C_9_ appear to be spontaneous while the *O*-acetyl group at C_4_ remains unaffected. 

*O*-Acetylation of Neu5Ac by SOATs is site-specific and biological functions are based on the position of acetyl groups. Much of the focus has revolved around *O*-acetylation at the C_7, 8,_ and _9_ positions of Neu5Ac due to high abundance across species [15,16,17], although C_4_-*O*-acetylated Neu5Ac has garnered more attention recently in the context of virus binding [30]. 

During viral infection, viral glycoproteins recognize Sia as receptor determinants to prompt entry and exit from the host cell [30]. Seen in certain virus species, recognition of sialoglycoconjugates with defined linkages and isoforms is apparent [33]. Hemagglutinin (HA) of influenza A and B viruses, for example, utilize α-2,3-linked sialoglycoconjugates for attachment [34,35], while hemagglutinin-neuraminidase glycoprotein of respirovirus can utilize α-2,6-linked sialoglycoconjugates for attachment. Meanwhile, coronaviruses, a positive-sense single stranded RNA virus classified under the family Coronaviridae, contain spike (S) glycoproteins (and hemagglutinin-esterases) that utilize *O*-acetyl forms of Sia for attachment [36,37,38]. Viral fusion with the host membrane promotes viral replication and release is dictated by receptor-destroying enzymes including sialidase (or neuraminidase) or hemagglutinin-esterases, depending on the virus type [30]. 

Viral pathogenicity, therefore, requires a specific host–receptor partnership. It was believed that Sia glycosylation patterns were the most critical features to virus binding; however, recognition of modified Sia appears to be equally important. More specifically, *O*-acetylation at varying sites of Sia (i.e. C_4_ vs. C_7–9_) influences virus recognition and attachment. Viral receptor molecules show remarkable specificity toward host sialic acid isoforms at a molecular level and delving deeper into the effects of *O*-acetylated Sia/viral enzymes may help identify a potential therapeutic target to prevent virus-related diseases.

## 2. Extraction and Detection Methods of *O*-Acetylated Neu5Ac

**Figure 3:**
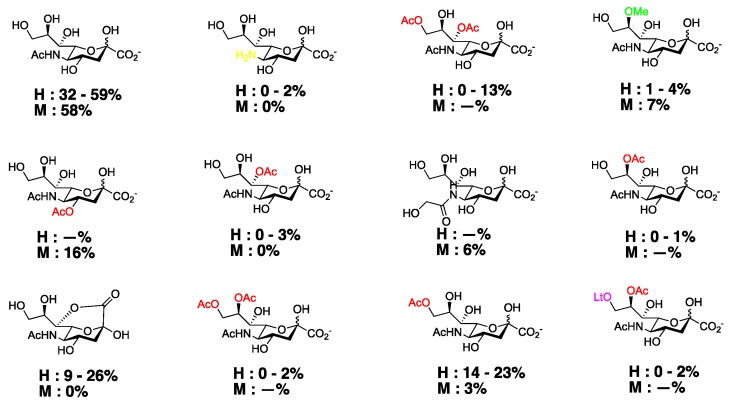
Distribution of isolated Neu5Ac analogs in human and mouse gut. H = humans, M = mice. Figure modified from [39].

Site-specific *O*-acetylated sialoglycoconjugates are found across a variety of species. C_9_ *O*-acetylated Neu5Acs, for instance, are found in the human gut ranging from 14–23% (Figure 3) [12,39]. Meanwhile, C_4_ *O*-acetylated Neu5Ac is ubiquitous in ungulates [11,22,40,41,42,43,44], echinoderms [45], Australian monotreme echidna [40], and a subgroup of chordates [42,43,44,46,47]. Among several vertebrates, C_4_-*O*-acetylated Neu5Ac was found mostly in the circulatory system of fish [47]. 

Enzymatic or mildly acidic hydrolytic release from mucins are conventional methods by which partially *O*-acetylated Neu5Ac have been extracted prior to characterization [48,49,50]. The most convenient isolation techniques involve the application of sialidases; however, issues related to manufacturing costs and activities without jeopardizing the integrity of *O*-acetyl groups prevent access to materials at a commercial scale. 

Among detection strategies, histochemical staining has become a widely accepted technique for detecting *O*-acetylated Neu5Ac with information on site-specificity (Table 2). Ravindranaths and co-workers, for instance, showed that radiolabeled, purified crab lectin from *Cancer antennarius* incubated with erythrocytes in Tris buffered saline bound to C_9_ *O*-acetylated Neu5Ac using radiolabelled assays [51]. More recently, advances in using matrix-assisted laser desorption/ionization mass spectrometry (MALDI) combined with imaging software have enabled mapping of the localization of *O*-acetylation on gangliosides [52,53]. Furthermore, modern techniques available for analyses include fluorescence detection by 1,2-diamino-4,5-methylenedioxybenzene [54], Neu5Ac analogs using high-performance liquid chromatography [55,56,57], colorimetric determination [58,59,60], thin-layer chromatography [60], electrospray-ionization mass spectrometry [61], GC-MS of silylated analogues [62], and NMR [53,63]. 

## 3. Biological Implications of C_7–9_ and C_4_ *O*-Acetylated Neu5Ac

**Figure 4:**
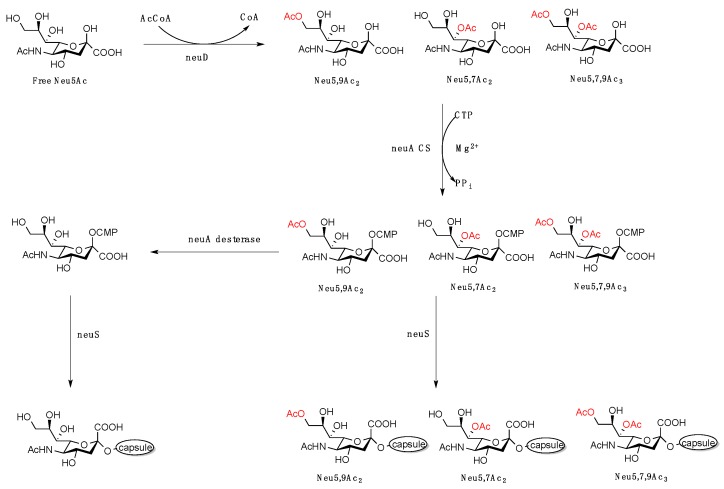
Bacterial enzymatic pathway for the biosynthesis of capsular Neu5Ac and capsular *O*-acetylated Neu5Ac. Figure modified from [99].

To the capsular polysaccharide (CPS) of *Streptococcus suis* that is responsible for causing meningitis, Jin and co-workers found that partially *O*-acetylated CMP Neu5Ac (e.g., Neu5,7(8,9)Ac_2_) are used as glycosyl donors rather than free CMP sialic acid [99]. Several strains of Gram-negative bacteria, such as *S. suis*, *E. coli*, *S. agalactiae,* modulate CPS by a bifunctional genetic code, NeuA, which encodes for CMP-Neu5Ac synthetase/*O*-acetylesterase activities (Figure 4). The reasons for which the bacteria utilize partially *O*-acetylated Neu5Ac as a substrate for CMP synthetase (CSS), only to be de-*O*-acetylated, are still under investigation. Nonetheless, this study suggests that *O*-acetylation of Neu5Ac is not exclusive to post-glycosylation modification. Rather, it can be used directly as substrates for glycosylation, although only the *O*-acetylated forms at the C_7–9_ were investigated. More recently, the Chen group demonstrated the feasibility of C_4_ *O*-acetylated Neu5Ac as substrates for CMP activation [100] using CSS from *Neisseria meningitidis* and mutants, *Pasteurella multocida*, and *Haemophilus ducreyi*. Upon CMP activation, PmST3 sialyltransferase was able to uptake CMP Neu4,5Ac_2_ to render α-2,3 linked sialoglycoconjugates without compromising the acetyl group.

Considering eukaryotic systems, *O*-acetylated Neu5Acs of colonic mucin glycoproteins from human colon cell lines are ubiquitous [37]. Corfield and co-workers showed the inhibition of *B. bifidum* VIII-210 sialidase activity by C_7,8,_ and _9_ *O*-acetylated Neu5Ac [101] in 1992. Interestingly, the inhibitory effects were increased as amounts of *O*-acetyl groups increased. When di-*O*-acetylated Neu5,7,9Ac_3_ was incubated with bacterial sialidases, sialic acid cleavage decreased two-fold compared to mono *O*-acetylated forms. Saponification conditions that de-*O*-acetylate led to an increase of released sialic acid by sialidases, which suggests that the presence of *O*-acetyl groups inhibits bacterial sialidase activities, although it remains unclear whether C_4_ *O*-acetylation exhibits similar functions. 

Since the discovery of *O*-acetylated Neu5Ac by Blix and co-workers [102], Pepper reported the effects of *O*-acetylated Neu5Ac on virus hemagglutinin/neuraminidase activities [103]. Concerning horse serum, C_4_-*O*-acetylated Neu5Ac accounts for approximately 50% of the total sialylation, as confirmed by structural elucidation using thin-layer chromatography, gas chromatography, and periodate oxidation. These studies revealed that the C_4_-*O*-acetylated Neu5Ac, found exclusively on α_2_-macroglubulin, inhibits hemagglutinin of the influenza A2 strain. Treatment with NaOH or NaIO_4_ gave slow to complete loss of hemagglutinin inhibition, presumably due to the loss of the C_4_ *O*-acetyl group [16,103]. Furthermore, Pepper was one of the first to demonstrate selective reactions to viral or bacterial neuraminidases by C_4_-*O*-acetylated Neu5Ac. This was a significant finding since it indicated that *O*-acetylated Sia determinants provide specific function (Table 3).

Added to the inhibitory effects of C_4_-*O*-acetylated Neu5Ac, it also has been implicated in facilitating the initial attachment of viruses to target cells. Similar to influenza C virus, infectious salmon anemia virus (ISAV) is classified under the Orthomyxoviridae family and contains spike glycoproteins hemagglutinin-esterase (HE) and fusion (HEF) proteins that mediate virus entry and exit. HE exhibits hemagglutination and receptor destroying activities and C_4_-*O*-acetylated Neu5Ac was defined as the major receptor determinant [81]. While influenza C virus recognizes C_9_-*O*-acetylated Neu5Ac, ISAV is selective for C_4_-*O*-acetylated Neu5Ac in receptor binding and destroying activities, despite known similarities to influenza C virus HE [51]. 

The receptor destroying enzyme displays acetylesterase activity and can cleave *O*-acetyl groups with high specificity. Regarding the case of ISAV, C_4_-*O*-acetyl was cleaved via 4-sialyl-*O*-acetylesterase with high turnover rates, while de-*O*-acetylation of C_9_-*O*-acetylated Neu5Ac required substantially extended incubation times. Furthermore, abolishment of hemagglutination inhibition was observed when guinea pig and horse sera were subjected to saponification conditions (e.g., 0.1 N NaOH) while rat serum exhibited no effects. Significantly, these studies provide evidence of molecular specificity depending on the position of acetyl groups (C_4_ vs. C_9_) and highlight the importance of enzymes that mediate these functions, which appear to be tailored to specific *O*-acetyl expression profiles of Sia. 

Seen in another case, toro- and corona-viruses of the Coronaviridae family display remarkable receptor binding specificity to *O*-acetylated Sia determinants. These viruses contain S and HE glycoproteins that mediate virus entry and exit. Similar to influenza C virus HEF, murine coronaviruses display HE activities consistent with the observation that this enzyme was acquired via horizontal gene transfer [136]. Thus, most of the murine coronavirus HEs recognize C_9_-*O*-acetylated Neu5Ac as Sia determinants; however, a subset of murine coronavirus HE failed to recognize C_4_-*O*-acetylated Neu5Ac. Specifically, HE of the mouse hepatitis virus (MHV) *DVIM*-strain recognizes C_9_-*O*-acetylated Neu5Ac, while HE of the MHV *S*-strain evolved to display strong binding preference toward C_4_-*O*-acetylated Neu5Ac ligand [83,85,140,141]. The crystal structure of MHV *DVIM*-HE closely resembles the overall architecture of coronavirus-Mebus (BCoV-Mebus) HE that favors C_9_-*O*-acetylated Neu5Ac as the binding ligand. While the R1-, R2-, and E-loops of MHV *S*-HE resemble BCov-Mebus HE, changes to the R3- and R4- loops are responsible for presenting preferential binding to one *O*-acetyl Sia form over another. Specifically, the hydrophobic pocket that once accommodated the C_5_ *N*- and C_9_ *O*-acetyl groups of Neu5Ac is mutated to accommodate the C_5_ *N*- and C_4_-*O*-acetyl groups of Neu5Ac. This conformational shift is responsible primarily for the change in the receptor specificity of MHV *S*- and *DVIM*-strains, although the reasons for such changes between distinct MHV lineages remain ambiguous [36,136]. The authors propose that evolutionary changes to accommodate the receptors that once remained neutral indicate plasticity of HE, which may lead to resistance against treatments.

Inhibition of sialidase activity by modified Sia also is involved in non-virus mediated phenomena. As a mechanism for survival, milk serves as a source of nutrients and bioactive components for offspring during early development; however, platypuses secrete milk through their skin, which poses a high risk for pathogenic infections [51]. Thus, platypuses produce *O*-acetylated Sia in elevated amounts, a variant of sialic acid shown to interfere with recognition by mammalian and bacterial sialidases. When glycoforms of Tasmanian echidna and platypus milk pools were analyzed [126], the milk oligosaccharides were found to be abundant in C_4_-*O*-acetylated sialyllactose, such that it comprised the majority of acidic oligosaccharides. Given that sialidase activity is inhibited by C_4_-*O*-acetylated Neu5Ac [129], the authors suggest that platypuses adopted this defensive strategy to counter-measure pathogenic mediated catabolism. 

## 4. Chemical Modifications of C_4_ Sia 

Site-specific *O*-acetylation of Sia imparts selective biological functions. Certain neuraminidases and/or esterase activities recognize *O*-acetyl groups on the C_4_ of Sia, but not at other positions. The presence of an acetyl group provides added sterics to block bacterial neuraminidases [103], while viral neuraminidase activities remain unaltered. These examples highlight the potential of modifying certain positions of Sia to induce a specific function. Von-Itzstein and co-workers developed in 1993 a rationally designed inhibitor against sialidases of influenza A and B viruses [142]. Aided by the X-ray crystallography and GRID calculations, it was determined that C_4_ substitution by a basic functional group would form a favorable interaction with Glu 119 in the active site. Given that sialidases bind to Sia in the boat conformation, modified Sia in the C_2_–C_3_ unsaturated form (Neu5Ac2en), bearing a guanidine group at the C_4_, inhibited sialidases of influenza A virus (Singapore/1/57) and B virus (Victoria/102/85) with IC_50_ values of 14 nM and 5 nM, respectively. This work was extended to inhibit sialidases of the human parainfluenza virus (hPIV) type 3 [143]. hPIV is classified under the family Paramyxoviridae that contains HN and fusion proteins. An X-ray crystal structure was deduced and the revelation of a hollow region near the C_4_ of Neu5Ac2en led to substituting various alkyl groups at the C_4_. Consequently, the *O*-ethyl group gave the best IC_50_ value of 3 μM, while zanamivir that contains the 4-guanidino group also inhibited hPIV-3 (strain C243) sialidases with an IC_50_ value of 25 μM. This further validates that inhibition of sialidases responds to specific groups of Neu5Ac2en at the C_4_ that have been rationally designed. Consistent with findings by Pepper [103], sialidase response to treatment appeared to be selective across species. These findings ultimately led to the development of oseltamivir (tradename Tamiflu, Basel, Switzerland) and zanamivir (tradename Relenza, London, UK) [144]. However, adverse effects and low efficacy led to decreased clinical use. Thus, efforts to develop inhibitors of sialidase as a therapeutic target are being investigated actively.

Current extraction methods give inadequate yields; thus, synthetic efforts to obtain *O*-acetylated Sias in pure form are valuable. Using a conventional synthetic approach, a series of mono-*O*-acetylated Neu5Ac have been synthesized, including Neu4,5Ac_2_, Neu5,8Ac_2_, Neu2,5Ac_2_, Neu5,7Ac_2_ [110]. These conjugates were synthesized in pure, monomeric forms without acetyl migrations or anomerization; thus, they serve as useful standards for delving deeper into studies related to partially *O*-acetylated Neu5Ac. More recently, a series of partially *O*-acetylated Neu5Ac have been synthesized using regioselective exchange technology (ReSET) by the Gervay–Hague laboratory [13]. Taking advantage of the reactivity at various silyl ethers on Neu5Ac, ReSET generated mono-, di-, tri-, and tetra-*O*-acetylated Neu5Ac in a site-specific manner. Notably, Neu4,5Ac_2_ was synthesized in four steps in a 19% yield with a 75% improved efficiency than the previous reported synthesis. Step-economical methodologies such as ReSET can offer access to a divergent library of *O*-acetylated Neu5Ac in an efficient and pure form. 

While chemical modifications of C_4_ Sia have been reported elsewhere [145,146,147,148], advances in esterase tolerant, synthetically efficient, sialidase inhibitors could lead to a novel therapeutic without losing efficacy. Substitution of the *O*-acetyl group at the C_9_ of Sia by an *N*-acetyl group minimized loss (or migration) of the acetyl group and retained a similar conformation as the *O*-ester. The authors argue that it can extend to other hydroxyl sites, including the C_4_ in principle, which can serve as *O*-acetylated Neu5Ac surrogates that may display significant sialidase inhibition [149]. 

Lead compounds can be generated using a rational drug design at the interface of synthetic chemistry, X-ray crystallography, and in silico studies. Particularly, blockades of Sia/sialidases hold tremendous therapeutic potential. Chemical modification at the C_4_ of Sia, therefore, represents a unique, bio-inspired Sia variant that may be tailored to increase therapeutic efficacy of antiviral molecules.

## 5. Conclusions

Modified Sias, specifically *O*-acetylated isoforms, have been recognized as receptor determinants of virus pathogenesis. *O*-Acetylation is the most common form of post-glycosylation modification of Sias in mammalian cells. Mediated by specific SOATs, *O*-acetylation can occur at the C_4_ or C_7–9_ of Sias in a highly regulated and well-defined manner. Yet, structural elucidation of *O*-acetylated Sias remains incomplete and its biological implications have only recently garnered greater attention. 

Modern extraction methods still remain a challenge to capture target molecules from a complex biological mixture in sufficient amounts. Methods to isolate site-specific *O*-acetylated sialic acids in high purity are needed. Given poor stability under current extraction techniques and spontaneous acetyl migrations that add to its structural complexity, synthetic access to *O*-acetylated Sias in high purity remains a valuable tool. 

The discovery of *O*-acetylated Sias as major receptor determinants of virus pathogenesis provides a unique opportunity to develop therapeutics. Specifically, modifications at the C_4_ may enhance sialidase inhibitor activities (Table 3). Using chemical methodologies such as ReSET can be extended to install chemical groups tailored to improve efficacy. Furthermore, chemical handling of C_4_ modifications would be much more feasible than C_7–9_ modifications, since intramolecular complications such as acetyl migrations are scarce. 

Different classes of viruses infect the host cell using glycoproteins (e.g., HA, HE, S, HEF) that aid in receptor binding or destroying functions. Most viral families, including Orthomyxoviridae, Paramyxoviridae, and Adenoviridae, utilize Sias on host membranes as recognition molecules for attachment and entry. However, only a subset of these human pathogens recognizes *O*-acetylated Sias, including influenza C, although studies related to this topic are currently under investigation. Moreover, recognition of *O*-acetylated isoforms appears to be site-specific. Taking this into account, C_4_ of Neu5Ac represents a unique position for chemical modifications that could enable lead discoveries.

## Figures and Tables

**Table 1:** Select examples of *O*-acetylated Neu5Ac found in Nature [12].

Sialic Acids	Occurrence ^a^
5-*N*-Acetyl-4-*O*-acetylneuraminic acid	V
5-*N*-Acetyl-7-*O*-acetylneuraminic acid	V, Pz, B
5-*N*-Acetyl-8-*O*-acetylneuraminic acid	V, B
5-*N*-Acetyl-9-*O*-acetylneuraminic acid	V, E, Pz, F, B, H [31]
5-*N*-Acetyl-4,9-di-*O*-acetylneuraminic acid	V
5-*N*-Acetyl-7,9-di-*O*-acetylneuraminic acid	V, B, H [32]
5-*N*-Acetyl-8,9-di-*O*-acetylneuraminic acid	V, H [32]
5-*N*-Acetyl-4,7,9-tri-*O*-acetylneuraminic acid	V
5-*N*-Acetyl-7,8,9-tri-*O*-acetylneuraminic acid	V
5-*N*-Acetyl-4,7,8,9-tetra-*O*-acetylneuraminic acid	V
5-*N*-Acetyl-9-*O*-lactylneuraminic acid	V
5-*N*-Acetyl-4-*O*-acetyl-9-*O*-lactylneuraminic acid	V
5-*N*-Acetyl-7-*O*-acetyl-9-*O*-lactylneuraminic acid	V
5-*N*-Acetyl-9-*O*-acetyl-8-*O*-methylneuraminic acid	V, E
5-*N*-Acetyl-4-*O*-acetyl-8-*O*-sulfoneuraminic acid	V, E
5-*N*-Acetyl-9-*O*-acetyl-2-deoxy-2,3-didehydroneuraminic acid	V
5-*N*-Acetyl-4,9-di-*O*-acetylneuraminic acid 1,7-lactone	V

^a^ Abbreviations used: V = vertebrates; H = humans; E.=.echinoderms; Ps = protostomes (insects and mollusks); Pz = protozoa; F = fungi; B = bacteria.

**Table 2:** Extraction and detection methods of *O*-acetylated Neu5Ac.

Conditions	+/−	Comments	References
Alkaline conditions	–	Acetyl migrations *(C_7_ and C_9_ OAc’s)*	[47,62]
Glycan release by NaOH	–	Loss of esters	[48]
Acidic hydrolysis with propionic acid vs. acetic acid	/	Loss of esters. Minimal if propionic acid (2M, 4 h, 80 °C) is used	[46,47,63,64]
Histochemical staining Mild periodic-Schiff	+	Stain after hydrolytic removal *(General method)*	[65]
Mild oxidation	+	Indirect quantitative determination by formaldehyde production *(For C_8_ and C_9_ OAcs)*	[66]
Lectin staining	+	from crab Cancer antennarius *(For C_9_ OAc)*	[49]
	+	from snail *Achatina fulica (For C_9_ OAc)*	[67,68,69,70]
Viral lectin staining	+	from influenza C *(For C_9_ and C_4_ OAc)*	[64,71,72,73,74,75,76,77,78,79,80]
	+	infection salmon anemia virus *(For C_4_ OAc)*	[81,82]
Coronavirus	+	Murine coronavirus *(For C_4_ OAc)*	[83,84]
		Porcine torovirus *(For C_9_ OAc)*	[85,86]
		Bovine torovirus *(For C_7(8)_, C_9_ OAc)*	[85]
		Hemagglutinating encephalomyelitis virus (*For C_9_ OAc*)	[87,88]
		Puffin coronavirus *(For C_4_ OAc)*	[84,89]
		Human coronavirus OC43 *(For C_9_ OAc)*	[90,91]
		Bovine coronavirus *(For C_9_ OAc)*	[85,92,93,94]
	+	Sialodacryo-adenitis virus *(For C_4_ OAc)*	[71]
		Monoclonal antibodies *(For OAc gangliosides)*	
	+	MAb Jones	[95]
	+	MAb UM4D4	[96]
	+	MAb U5	[97]
	+	MAb 7H2	[98]
MALDI mass spectrometry imaging	+	*(For OAc gangliosides)*	[50,51]

+ = good/acceptable method; – = poor method and results in complications.

**Table 3:** Compilation of relevant studies associated with C_4_ *O*-acetylated Neu5Ac.

Topic	References
Chemical synthesis	
Synthesis of Neu4,5Ac_2_ and Neu4,5,9Ac_3_ methylate	[104]
Synthesis of Neu4,5Ac_2_ with methylcoumarin	[105]
Synthesis of fluorescent 4-*O*-acetyl thioketosides Neu5Ac	[106]
Synthesis of 4-*O*-acetyl ketosides of Neu5Ac	[107]
Synthesis of 4-*O*-acetyl containing GM3s	[108]
Synthesis of Neu4,5Ac_2_; Neu4,5,9Ac_3_; Neu4,5,8,9Ac_4_; Neu2,4,5,8,9Ac_5_; Neu4,5,7,8,9Ac_5_	[13,109,110]
Structural analysis	
Identification of Neu4,5Ac_2_, Neu5,9Ac_2_, Neu4,5,9Ac_3_, Neu5,7,9Ac_3_, Neu4Ac_5_Gc, Neu9Ac_5_Gc by mass spectrometry	[60]
NMR studies on Neu4,5Ac_2_(α2→3) lactose	[38]
FAB-MS analysis of C_4_-*O*-acetylated Neu5Ac	[111]
Characterization of *O*-acetylated GM3s in equine erythrocytes	[112]
Computational/conformational studies on Neu4,5Ac_2_	[113]
Molecular dynamics studies on C_4_-*O*-acetylated Neu5Ac on hemagglutinin activity and its receptor binding site	[114]
Oxidative studies	
Periodate oxidation on Neu4,5Ac_2_ studies	[104]
Periodate oxidation on Neu4,5Ac_2_ of murine erythrocyte ghosts	[115]
Periodate oxidation on *O*-acetyl sialosides from rat salivary glands	[116]
Isolation of C_4_-*O*-acetylated containing Neu5Ac from various sources	
Isolation from gangliosides	[117]
Isolation from colonic epithelial cells	[118]
Isolation from hamster sublingual gland	[119]
Isolation from murine erythrocyte ghosts (DBA/2, CD-1, B6D2 strains)	[115]
Isolation from bovine and equine submaxillary mucins	[120]
Isolation from influenza C	[121]
Isolation from salmon eggs	[122]
Isolation from equine erythrocytes	[112]
Isolation from rat submandibular glands	[116]
Isolation of 4-*O*-Ac-GM3 from equine erythrocytes	[117]
Isolation from starfish A. *rubens*	[43]
Isolation from vertebrates (fishes)	[45]
Acid/Enzyme hydrolysis from rabbit urine glycoprotein	[123]
Isolation of *O*-acetylated Neu5Ac using propionic acid	[63]
Isolation from rat coronavirus	[71]
Hydrolysis from murine coronaviruses in mouse tissue	[124]
*O*-Acetylesterases from horse liver specific for Neu4,5Ac_2_	[125]
Hydrolysis from infectious salmon anemia virus (ISAV)	[81]
Isolation from echidna and platypus milk	[51,126]
Inhibitory activities of C_4_-*O*-acetylated containing Neu5Ac	
A2 viral strain inhibited by horse serum containing Neu4,5Ac_2_	[103]
Neuraminidase from *S. sanguis* cannot cleave C_4_-*O*-acetylated Neu5Ac	[127]
Sialidase from human leukocytes cannot cleave C_4_-*O*-acetylated Neu5Ac	[128]
Sialidase from C. *perfringens* cannot cleave C_4_-*O*-acetylated Neu5Ac	[129]
Bacterial sialidase activities inhibited by C_4_-*O*-acetylated Neu5Ac	[130]
Inhibition of rabbit red blood cell agglutination activities by Achatinin_H_	[67]
Inhibition of hemagglutinin activities of lectin bindings in human placenta	[131]
Influenza viruses (H3N2 strain) binding to Neu4,5Ac_2_ while unrecognized by B or H1N1 viruses	[114]
Viral inhibitory effects by equine and pig sera based on Neu4,5Ac_2_ located on the α2 macroglobulins	[132]
Esterase hydrolysis of acetyl groups	
Acetylesterases from horse release Neu4,5Ac_2_	[133]
ISAV esterases bind and hydrolyze C_4_-OAc Neu5Ac	[81]
C_4_-OAc Neu5Ac is the preferred binding receptor of ISAV	[44]
Metabolism of partially O-acetylated Neu5Ac from bovine and equine submandibular glands	[134]
4-*O*-Acetylated Neu5Ac in equine and guinea pig α2 macroglobulins	[39]
*O*-Acetyltransferase in C_4_-OAc Neu5Ac biosynthesis	[21]
*O*-Acetylation on C_4_-OAc Neu5Ac	[42]
Mouse hepatitis virus S esterase cleaves C_4_-OAc Neu5Ac	[83]
Binding studies on C_4_-*O*-acetylated Neu5Ac	
Micronemes show strong binding preference to C_4_-OAc Neu5Ac	[135]
Mouse hepatitis virus S binds and recognizes C_4_-OAc Neu5Ac	[136]
Infectious Salmon Anaemia Virus (ISAV) binds and recognizes C_4_-OAc Neu5Ac	[137,138,139]
Crab (*Cancer antennarius*) lectin binds to C_4_-OAc Neu5Ac	[49]
